# Supplementary material for: Tuning Ising superconductivity with layer and spin–orbit coupling in two-dimensional transition-metal dichalcogenides
Source: Nat Commun. 2018 Apr 12;9:1427. doi: 10.1038/s41467-018-03888-4 (PMC5897486; doi:10.1038/s41467-018-03888-4)
Supplement: Supplementary file 1 — Supplementary Information [file 41467_2018_3888_MOESM1_ESM.pdf]

# Tuning Ising superconductivity with layer and spin-orbit coupling in two-dimensional transition-metal dichalcogenides

de la Barrera *et al.*

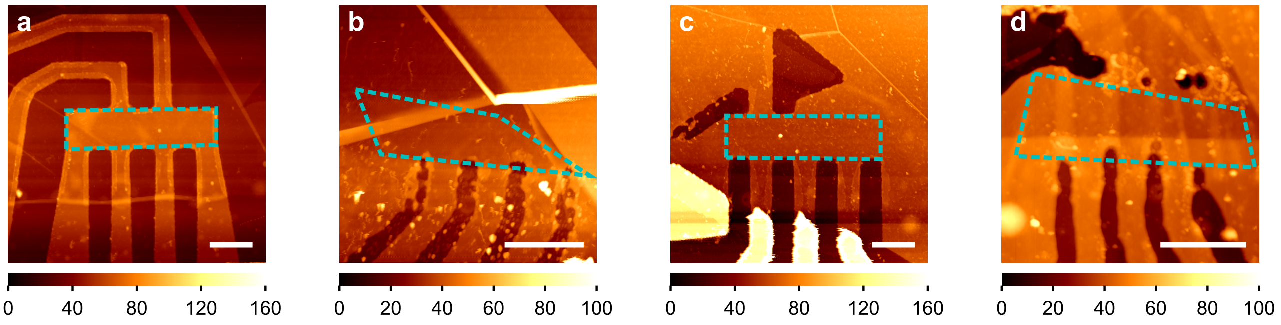


**Supplementary Figure 1**. **Atomic force microscopy of $TaS_2$ devices.** Height scans showing the lithographically defined $TaS_2$ channels (dotted region) using non-contact mode atomic force microscopy for (a) five-layer, (b) four-layer, (c) trilayer, and (d) monolayer devices. The total height of each channel primarily composed of a relatively thick ($\approx 20\,\mathrm{nm}$) h-BN encapsulating layer protecting the thin, underlying $TaS_2$ crystal. Scale bars denote $2\,\mu\mathrm{m}$. Color scales correspond to height measured in nanometers.

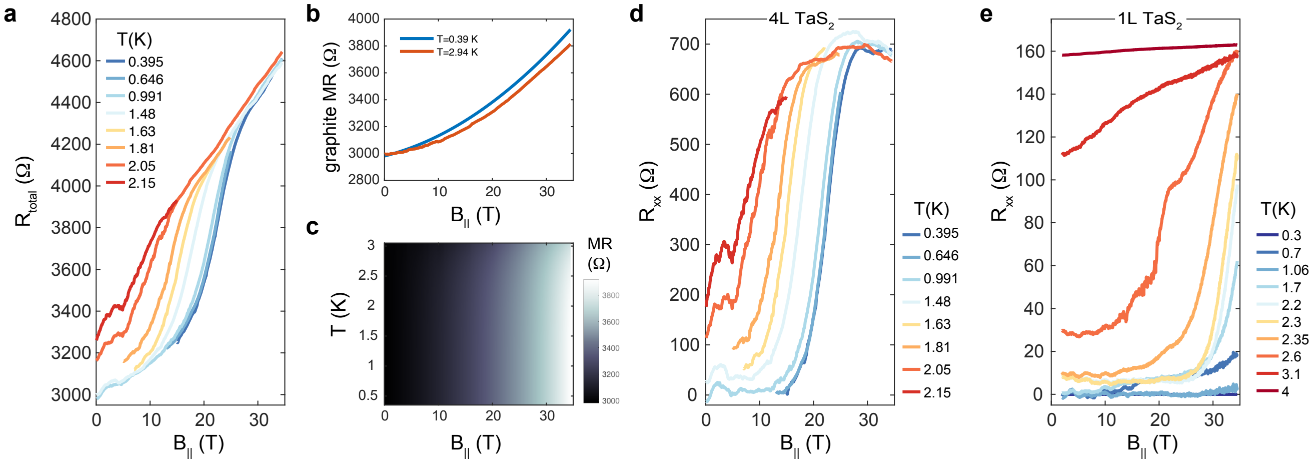


**Supplementary Figure 2**. **Procedure for background subtraction of graphite magnetoresistance.** (a) Three-terminal measurement of $R_{\mathrm{total}}$ for the 4L $TaS_2$ device, which includes a background magnetoresistance $R_{\mathrm{graphite}}$ from the graphite contacts. (b) High-$T$ (2.94 K) and low-$T$ (0.39 K) curves used to generate background $R_{\mathrm{graphite}}(B_{\|}, T)$. The high-$T$ curve (2.94 K) has the normal state resistance $R_n \approx 700\Omega$ subtracted; the low-$T$ curve (0.395 K) was generated by fitting a polynomial to the 0.395 K data from (a) for $B < 12\,\mathrm{T}$. (c) Surface of $R_{\mathrm{graphite}}(B_{\|}, T)$ generated by interpolating between the two curves from (b). (d) Subtracted $R(B_{\|})$ curves at various $T$ for 4L $TaS_2$, which are used to extract $H_{c2}^{\|}(T)$. Temperature legend is same as in (a). (e) Subtracted $R(B_{\|})$ curves at various $T$ for 1L $TaS_2$, generated by an identical subtraction procedure as described for the 4L device.

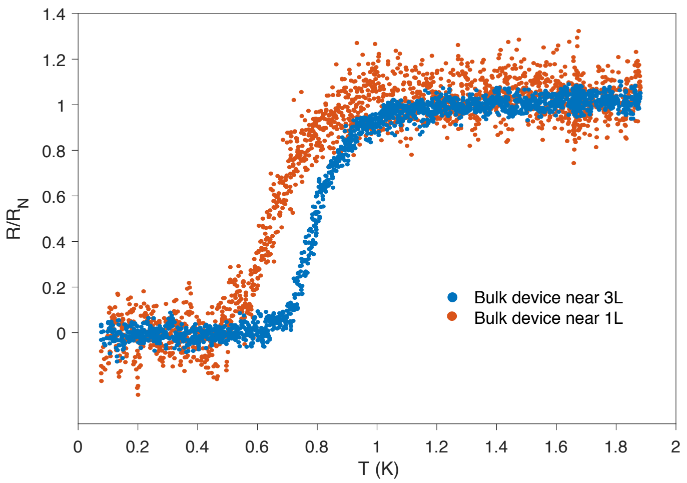


**Supplementary Figure 3**. **Confirmation of bulk $TaS_2$ properties.** Temperature dependence of bulk $TaS_2$ sheet resistance for two-terminal devices fabricated using flakes $\gg 20\,\mathrm{nm}$ thick, near the 1L and 3L few-layer devices on each substrate.

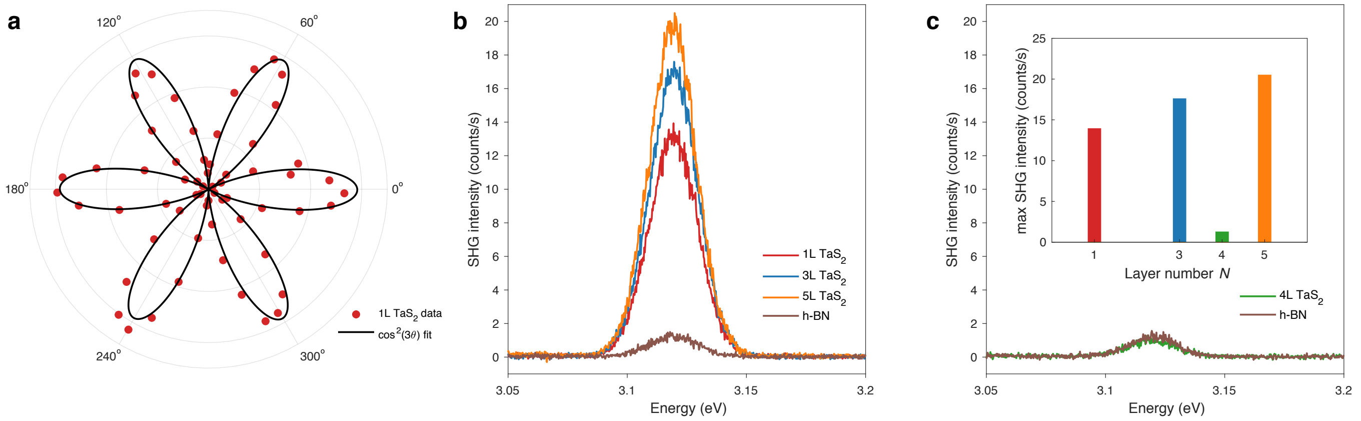


**Supplementary Figure 4**. **Second harmonic generation of $TaS_2$ devices.** (a) Polar plot of co-linear second harmonic generation (SHG) intensity (spectrally-integrated) as a function of incident polarization angle from monolayer 1H-$TaS_2$. (b) SHG spectra for three odd layer number $TaS_2$ devices with h-BN spectrum for comparison. (c) SHG spectrum for an even layer number $TaS_2$ device with h-BN spectrum for comparison. Inset: maximum SHG intensity observed for the four samples of various thickness.

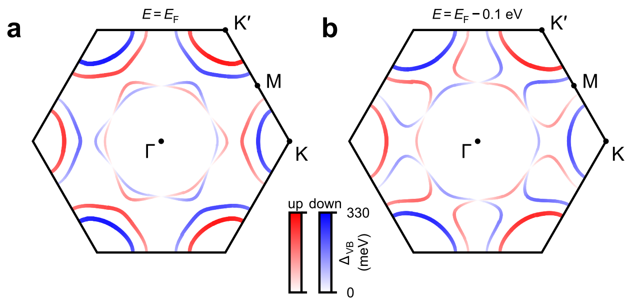


**Supplementary Figure 5**. **Qualitative change in $TaS_2$ Fermi surface upon lowering the Fermi level.** Fermi surface of monolayer $TaS_2$ computed by density functional theory using (a) the intrinsic Fermi level and (b) an adjusted Fermi level $0.1\,\mathrm{eV}$ lower than the intrinsic case, below the saddle-point along Γ–K of the upper spin band.

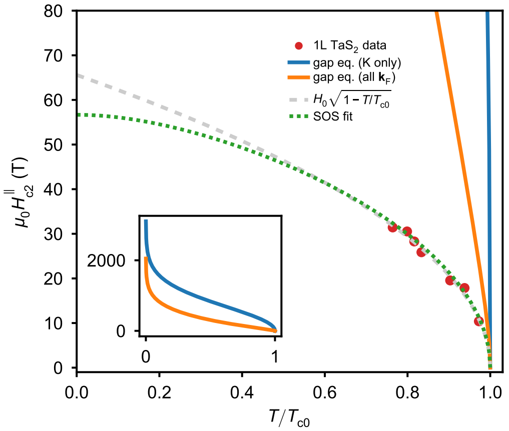


**Supplementary Figure 6**. **Theoretical upper critical line of 1L $TaS_2$.** Measured upper critical field of 1L $TaS_2$ reproduced from main text with phenomenological fits (dashed curves) and density functional theory (DFT) predictions (solid curves). DFT predictions result from solution of Supplementary Eq. (1) around the K, K′ pockets (K only) and from including the entire Fermi surface (all $\mathbf{k}_\mathrm{F}$), as indicated. Inset shows full scale of predicted curves in matching units. Sqaure root fit arises from 2D Ginzburg–Landau theory, as described in main text, with $H_0 = 65.6\,\mathrm{T}$. Spin-orbit scattering (SOS) fit results from solution of Supplementary Eq. (2) with $\tau_\mathrm{so}$ as a free parameter. Best fit $\tau_\mathrm{so} = 9.30\,\mathrm{fs}$.

## Supplementary Note 1: Characterization of devices with atomic force microscopy

To prevent $TaS_2$ and $NbSe_2$ crystals from degrading due to air exposure, we encapsulated all of our devices with an insulating layer of h-BN. Nevertheless, it is important to inspect the interface between the h-BN and superconducting layer to determine both the cleanliness of the interface (from trapped residues) and the degree of protection against degradation provided by the h-BN. We therefore measure the surface height variation with atomic force microscopy (AFM) after magnetotransport measurements for each device (Supplementary Figure 1). From the AFM images, we find that there are no pockets of trapped gas or fabrication residues, bubbles, and only one or two particles taller than $> 5\,\mathrm{nm}$ trapped between the h-BN and superconductor in the channel of each of our devices. We find that the root-mean-square roughness of the superconducting channels (dashed regions in Supplementary Figure 1a–d) are $0.7\,\mathrm{nm}$, $1.3\,\mathrm{nm}$, $0.4\,\mathrm{nm}$, and $0.3\,\mathrm{nm}$ for the 5L, 4L, 3L, and 1L $TaS_2$ devices shown in Fig. 4 of the main text, respectively. Although h-BN encapsulation does appear to protect the interior region of each crystal for a period of days or weeks with limited time in air, the AFM images do show signs of degradation near any etched edges, as can be seen along the borders of each electrical lead in Supplementary Figure 1. The degraded edges appear in all etched edges of $TaS_2$, but do not appear along the etched edges of graphite regardless of the time spent in air. Degradation beginning along the edge can eventually creep into the interior and prevent superconductivity (especially in the narrow lead regions), as was observed in the 1L $TaS_2$ device after a period of two months.

## Supplementary Note 2: Background subtraction on three-terminal devices

In two of our devices (1L and 4L $TaS_2$), broken contacts forced us to measure $R_\mathrm{total}(B_\|, T)$ in a three-terminal geometry (e.g. Supplementary Figure 2a). This meant that we could not eliminate one of the contact resistances from our measurement, each contact consisting of graphite in series with Cr/Pd/Au (see Methods). Because of the temperature-dependent, strong magnetoresistance (MR) of graphite, the procedure used to subtract the background was non-trivial. We describe it here.

Without a direct measurement of the MR of graphite as a function of $B_\|$ and $T$, we generate a surface $R_\mathrm{graphite}(B_\|, T)$ by interpolating between two curves. Starting with each $R(B_\|, T > T_c)$ curve, where the total resistance is the graphite MR at $T > T_c$ plus a constant offset ($R_\mathrm{n}$ of the superconductor in the normal state, with negligible MR), we subtract $R(B_\|, 0)$, where the device is superconducting for low fields. For the latter, a reasonable guess can be generated for $R_\mathrm{graphite}(B_\|, 0)$ by fitting the low-field portion of $R_\mathrm{total}(B_\|, 0)$ to a polynomial, or in the case of the 1L $TaS_2$ data, by simply using the $R_\mathrm{total}(B_\|, 0)$ curve as $R_\mathrm{graphite}(B_\|, 0)$ since the device does not go normal for any field in this limit.

Since the background curves are relatively insensitive to temperature (Supplementary Figure 2b–c), the values of $H_\mathrm{c2}^\|(T)$ extracted from this procedure are quite insensitive to precisely how the interpolation was performed. For example, we can

estimate that difference in the value of $H_{c2}^{\parallel}(T)$ extracted directly from the raw data differs from that extracted by a linear interpolation of $R_{\mathrm{graphite}}(B_{\parallel}, 0)$ by at most $\pm 1\,\mathrm{T}$. A similar conclusion can be drawn for the difference in $H_{c2}^{\parallel}(T)$ deduced from a linear interpolation versus an exponential interpolation of $R_{\mathrm{graphite}}(B_{\parallel}, 0)$.

## Supplementary Note 3: Control experiments on bulk devices

The superconducting properties of $TaS_2$ (as well as other TMD superconductors) are known to change in the presence of intercalants or some non-stoichiometric mixtures [1–3]. In our studies, bulk $TaS_2$ crystals were formed using chemical vapor transport (from HQ Graphene). The stoichiometry was determined from energy-dispersive x-ray spectroscopy (EDX) to be $67.2\,\%$ sulfur and $32.8\,\%$ tantalum for one bulk crystal and $69.6\,\%$ sulfur and $35.5\,\%$ tantalum for another, indicating a slight excess of sulfur. Excess sulfur is not known to affect the superconducting properties of $TaS_2$ (whereas excess Ta is known to raise $T_c$ in some cases [1, 4]). Nevertheless, to exclude this possibility for the data presented in Figs. 2–4 for the 1L and 3L samples, we fabricated quasi-bulk devices from thick crystals ($\gg 20\,\mathrm{nm}$) exfoliated simultaneously with our atomically-thin crystals, onto the same substrate, and subject to the same fabrication process (with the exception of encapsulation by h-BN/graphite contacts). The two bulk devices exhibited much lower $T_c$ than the thin devices on the same substrate (Supplementary Figure 3), comparable to the expected value of the bulk $T_c = 0.5\,\mathrm{K}$ to $0.8\,\mathrm{K}$, which provides evidence that the enhancement of $T_c$ in the thin devices (and therefore also the enhancement of $H_{c2}^{\parallel}$) is indeed due to the reduction of $N$. An additional piece of evidence supporting this idea is the discrepancy between the shape of the $H_{c2}^{\parallel}(T)$ curves for intercalated $TaS_2$, which have a finite slope $dH_{c2}^{\parallel}/dT$ and upward curvature at $T_{c0}$, and are generally well-fit by the Klemm-Luther-Beasley theory [5]. Our monolayer and few-layer $TaS_2$ devices exhibit very steep (infinite) derivative at $T_{c0}$, showing downward curvature and no inflection point as $T$ is lowered.

## Supplementary Note 4: Second-harmonic generation study of $TaS_2$ devices

Second-harmonic generation (SHG) is a sensitive probe of inversion symmetry breaking, since the second-order nonlinear susceptibility is only nonzero for noncentrosymmetric crystals [6]. In few-layer 2H-phase TMDs, strong SHG is only observed in samples with odd layer number, where inversion symmetry is broken [7]. We studied the SHG from our $TaS_2$ samples to clearly distinguish between even and odd layer number, and thus further corroborate our sample thickness assignments. The samples were excited by a mode-locked Ti:sapphire laser (76 MHz repetition rate, 200 fs pulse duration, 1 mW average power) at 795 nm under normal incidence in reflection geometry and the SHG was detected by a spectrometer and Si charge-coupled device. The measurements were performed at room temperature under vacuum, and laser intensity was carefully tuned to prevent damage to the TMD material. In Supplementary Figure 4, we show the SHG polarization dependence and spectra from $TaS_2$ samples. The monolayer $TaS_2$ response to rotating the incident polarization forms a 'rose' pattern and is shown in Supplementary Figure 4a. The spectra of three odd layer number samples (1L, 3L, and 5L) are shown in Supplementary Figure 4b, while the spectrum of an even layer number (4L) sample is shown in Supplementary Figure 4c. It can clearly be seen that the SHG intensity of the odd layer number $TaS_2$ samples is significantly greater than that of the thin bulk h-BN that encapsulates them. On the other hand, the even layer number $TaS_2$ sample produces an intensity nearly equivalent to that of the encapsulating h-BN layer, showing that the 4L $TaS_2$ does not contribute significantly to the SHG response. The inset of Supplementary Figure 4c shows the maximum intensity observed for each of the four samples of varying thickness, demonstrating an oscillation in maximum intensity between odd and even layer number samples, similar to other 2H-phase TMDs [7]. Although a linear increasing trend is observed among the odd layer number samples, we draw no conclusion regarding this trend because of the small sample size. The even and odd layer number assignments established by SHG are consistent with the thickness determined by transport and AFM.

## Supplementary Note 5: Potential for spin-texture tunability in the Fermi surface of monolayer $TaS_2$

The intrinsic Fermi level of 1H-$TaS_2$ as computed by DFT is within $32\,\mathrm{meV}$ of a saddle point along $\Gamma$–K in the upper spin band, as shown in Fig. 1e. Two additional saddle points occur in a larger energy window: one from the lower spin band at the same location in $\mathbf{k}$-space and one at the spin-degenerate M point, $234\,\mathrm{meV}$ and $278\,\mathrm{meV}$ below the intrinsic Fermi level, respectively. Each of these saddle points is associated with a divergence in the density of states resulting from a van Hove singularity. Tuning the Fermi level in a monolayer of $TaS_2$ to coincide with any of these saddle points might therefore be

expected to produce interesting consequences for the superconductivity, for example, producing additional enhancement in the $T_{c0}$ due to the divergent density of states. Such tuning may be possible via doping or electrostatic gating techniques as employed in other TMD superconducting materials [8–13]. Furthermore, tuning the Fermi level to lie between the two saddle points along $\Gamma$–K would lead to a complete spin-polarization of the states along the $\Gamma$–K line of symmetry within each irreducible wedge of the Brillouin zone (Supplementary Figure 5). As shown in Supplementary Figure 5b, lowering the Fermi level by as little as $100\,\mathrm{meV}$ would be sufficient to qualitatively alter the Fermi surface geometry from the intrinsic case. Such a spin configuration in momentum space may be favorable for unconventional superconducting pairings such as chiral or spatially-modulated states [14].

## Supplementary Note 6: Additional comments on layer dependence of $T_c$

The critical temperature in bulk $TaS_2$ is known to increase from the intrinsic value $T_{c0} \approx 0.8\,\mathrm{K}$ to as high as $6\,\mathrm{K}$ when intercalated with various organic molecules, experiencing an associated increase in interlayer spacing and exhibiting anisotropic critical field behavior [2] indicative of a dimensional crossover from 3D to 2D, as described by the theory of Klemm, Luther, and Beasley [5]. The exact origin of this enhancement is not fully understood, but the suppression of competing charge-density-wave (CDW) order has been suggested to contribute in intercalated $TaS_2$ and other TMD layered compounds [3]. Evidently, a $T_{c0}$ enhancement up to $3\,\mathrm{K}$ was also observed in randomly-stacked $TaS_2$ nanosheets with a random twist angle between the layers, and no CDW transition was observed in the twisted stacks from $2\,\mathrm{K}$ to $300\,\mathrm{K}$ [15]. Epitaxial 1H-$TaS_2$ formed on Au(111) substrates also does not exhibit a CDW when measured directly by scanning tunneling microscopy at $4.7\,\mathrm{K}$ [16], although the authors note that the most likely explanation is possible interface doping from the Au substrate. On the other hand, an unexpected *coexisting* CDW phase was recently shown to persist along with enhanced superconductivity in bulk $TaS_2$ under increased pressures up to at least $25\,\mathrm{GPa}$, with an associated increase in $T_{c0}$ up to $8.5\,\mathrm{K}$ at $9.5\,\mathrm{GPa}$ [17], which seems to suggest that competition between CDW and superconducting order in these materials may not be universally applicable. In another proposal, the enhancement of $T_{c0}$ in $TaS_2$ thinned down to $3.5\,\mathrm{nm}$ is suggested to result from a strong, repulsive Coulomb interaction that is renormalized as the number of layers is reduced [18]. Finally, in a recent theoretical study of several thin-film type-II superconductors with thicknesses less than the out-of-plane coherence length, a rather general model for the opening of a second superconducting gap that coexists with the bulk gap was linked to an enhancement of $T_{c0}$ [19]. Although we find that the increasing trend in atomically-thin $TaS_2$ continues to the monolayer limit, we cannot distinguish between the aforementioned mechanisms based on our magnetotransport experiments.

## Supplementary Discussion

In monolayer TMDs, the strong Ising SOC pins the electron spins to the out-of-plane direction (Fig. 1b and Fig. 4c in main text), reducing the spin susceptibility to in-plane fields relative to the Pauli paramagnetic case, $\chi_n^{\parallel} < \chi_{\mathrm{P}}$. In the Discussion of the main text and in Ref. 20, the enhancement of $H_{c2}^{\parallel}$ above the Pauli limit in 1L $TaS_2$ and 1L $NbSe_2$ is interpreted as arising from this modified (van Vleck) in-plane susceptibility. This analysis suggests a clear scaling of the upper critical field with the strength of the SOC for the metallic TMDs, as we might expect. However, this scaling does not appear to extend to the measured upper critical fields of superconducting electrolyte-gated $MoS_2$ and $WS_2$ [9, 11, 13]. In particular, the upper critical fields of $MoS_2$ and $NbSe_2$ (normalized to $H_{\mathrm{P}}$) are quite similar, despite the valence band splitting in $NbSe_2$ due to SOC being at least $4\times$ larger than the conduction band splitting around the K pocket in $MoS_2$. To explain this behavior in $MoS_2$, Lu *et al.* [9] evaluate a linear gap equation including both Ising and Rashba SOC (the latter is negligible in our system) and compute the upper critical line for a vanishing gap. The same linear gap equation was originally derived for singlet pairing in noncentrosymmetric superconductors by Frigeri *et al.* [21] for general, antisymmetric $\mathbf{k}$-dependent SOC. Applying this model to our system, with purely Ising SOC, an in-plane external field, and a trivial singlet order parameter, the upper critical line is described by

$$\ln\frac{T}{T_{c0}} = \left\langle \frac{b^2}{\rho_{\mathbf{k}}^2}\,\mathrm{Re}\left[\psi\left(\frac{1}{2}\right) - \psi\left(\frac{1}{2} + \frac{i\rho_{\mathbf{k}}}{2\pi k_{\mathrm{B}}T}\right)\right]\right\rangle_{\mathbf{k}_{\mathrm{F}}}, \tag{1}$$

where $b = \mu_{\mathrm{B}} H_{c2}^{\parallel}$, $\rho_{\mathbf{k}} = \sqrt{b^2 + \Delta_{\mathrm{vb}}^2(\mathbf{k})}$, and $\langle\ \rangle_{\mathbf{k}_{\mathrm{F}}}$ denotes the average over the Fermi surface. However, in the absence of a competing effect such as Rashba SOC (which tends to tilt the spins in plane) the predicted transition curve vastly overestimates the measured upper critical line. For example, using the DFT-computed spin splitting around the K, K′ pockets of the 1L $TaS_2$ Fermi surface (shown in Fig. 1c in main text), the upper critical line determined by solving Supplementary Eq. (1) is over $15\times$ larger than experiment for the available range of data (K only curve in Supplementary Figure 6), and diverges as $T \to 0$. As discussed in the main text, the $\Gamma$ pocket of the Fermi surface also contributes to superconductivity in the metallic (group-V) TMDs, therefore we repeat the calculation including contributions from the K, K′, and $\Gamma$ pockets and find that the overestimation is partially reduced, but still significantly larger than experiment (all $\mathbf{k}_{\mathrm{F}}$ curve in Supplementary Figure 6).

In the low-temperature limit, the upper critical field in Eq. (1) diverges, $H_{c2}^{\parallel} \to \infty$ as $T \to 0$ (as shown in inset of Supplementary Figure 6). This prediction is consistent with the energy balance given in Eq. (1) of the main text, since our normal state susceptibility to parallel fields is reduced to a van Vleck susceptibility, $\chi_n^{\parallel} = \chi_{\mathrm{vV}} \propto \Delta_{\mathrm{so}}^{-1}$, and the van Vleck susceptibility persists into the superconducting state, $\chi_s^{\parallel} \to \chi_{\mathrm{vV}}$, even as $T \to 0$ [22]. Although the low-temperature behavior of $H_{c2}^{\parallel}$ for monolayer $TaS_2$ and $NbSe_2$ is beyond the range of available fields in these experiments, it is notable that the low-temperature dependence for few-layer samples (which is accessible in experiment) does not exhibit a trend toward diverging $H_{c2}^{\parallel}$.

Another important consequence of antisymmetric SOC (and thus Ising SOC) is an induced fraction of spin-*triplet* Cooper pairing [21, 23, 24]. Strong antisymmetric SOC (compared to the bandwidth) causes the suppression of interband pairing between upper and lower spin-orbit bands, resulting in an admixture of spin-singlet and spin-triplet pairing in each band [24, 25]. Ignoring for the moment the likely mixed-parity nature of pairing states in $TaS_2$ and $NbSe_2$, for decoupled singlet and triplet pairing, the triplet channel would posses a separate gap and paramagnetic limit. Also in the limit of strong SOC, the triplet order parameter $\mathbf{d}(\mathbf{k})$ is forced by symmetry to lie parallel to the SOC field $\mathbf{d}(\mathbf{k}) \parallel \mathbf{B}_{\mathrm{so}}(\mathbf{k})$ [21]. However, as shown by Frigeri *et al.* [21], for an external field perpendicular to the triplet order parameter for all $\mathbf{k}$ (indeed the case for in-plane field and out-of-plane Ising SOC), paramagnetic limiting is entirely absent. With this in consideration, the disagreement between the predicted upper critical line and experiment may in fact be larger still than what is shown in Supplementary Figure 6 including only singlet pairing, since a parallel magnetic field cannot break the fraction of triplet Cooper pairs with parallel spins [25].

Given that the models described so far are applicable in the clean limit, it is worth considering the opposite limit in which an extrinsic process may be in fact limiting the enhancement of the upper critical field. We are not able to estimate the mean free path of our 1L $TaS_2$ data, however we do have Hall measurements of the 5L sample, yielding a Drude mean free path of $l \approx 1.4\,\mathrm{nm}$. From our 2L $NbSe_2$ sample, we find $l \approx 17\,\mathrm{nm}$. Comparing these values to the Ginzburg–Landau coherence lengths from our perpendicular field measurements ($\approx 20\,\mathrm{nm}$ and $\approx 10\,\mathrm{nm}$, respectively) we see that while these materials are not unequivocally in the dirty limit of superconductivity ($l \ll \xi_0$), they are potentially in an intermediate regime. We therefore offer two alternative interpretations arising from spin-orbit and intervalley scattering.

Allowing for the possibility that the materials studied here may include sufficient disorder to affect the superconductivity, we may alternatively interpret the enhancement of $H_{c2}^{\parallel}$ in terms of a relevant scattering mechanism, such as spin-orbit scattering (SOS). In this limit, the spin-randomizing effect of SOS leads to a simple equation for the upper critical field,

$$\ln\frac{T}{T_{c0}} = \psi\left(\frac{1}{2}\right) - \psi\left(\frac{1}{2} + \frac{\alpha}{2\pi k_{\mathrm{B}}T}\right) \tag{2}$$

with $\alpha = 3\tau_{so}(\mu_B H)^2/2\hbar$, physically associated with a spin-orbit scattering time $\tau_{so}$, the average time between spin flips due to SOC with a scattering process [5]. This process is well-known for producing an upper critical field above the Pauli limit in a range of bulk and intercalated TMD superconductors [2]. Fitting this equation to the monolayer data in Fig. 4a–b, we find $\tau_{so} = 9.30\,\text{fs}$ and $\tau_{so} = 22.2\,\text{fs}$ for 1L $TaS_2$ and 1L $NbSe_2$, respectively (fit shown for 1L $TaS_2$ in Supplementary Figure 6). These SOS times are lower (as expected for larger $H_{c2}^{\parallel}$), yet comparable to measurements of intercalated bulk $TaS_2$ (20 fs to 150 fs [2]) as well as quasi-2D superconducting $MoS_2$ (20 fs to 40 fs [9]).

Separate from SOS, intervalley scattering (i.e. from the K to the K′ valley or vice-versa) requires a spin-flip and thus may contribute to pair-breaking in the superconducting state. Ilić *et al.* [26] generalized the model of Frigeri *et al.* [21] for computing $H_{c2}^{\parallel}$ (as we have expressed in Supplementary Eq. (1)) to arbitrary disorder, including intra- and intervalley scattering (only the latter breaks time-reversal symmetry and thus contributes to pair-breaking). The detailed result is beyond the scope of this paper, however we may find some utility in evaluating the strong SOC, intermediate disorder limit in which our system may belong. Specifically, in the $T \to T_{c0}$ limit,

$$H_{c2}^{\parallel}(T) \approx \frac{2\Delta_{so}}{\mu_B}\sqrt{\frac{\tau_2 k_B T_{c0}}{\pi\hbar}(1 - T/T_{c0})}, \tag{3}$$

where $\tau_2$ is the intervalley scattering time. In this limit, the square-root dependence allows direct comparison with the 2D Ginzburg–Landau fit described in the main text (fit shown in Fig. 4a–b and Supplementary Figure 6). Using the fit coefficient for 1L $TaS_2$ from the main text, $H_0 = 65.6\,\text{T}$, and an estimate of the SOC strength from the DFT average over the full Fermi surface $\Delta_{so} = \langle\Delta_{vb}(\mathbf{k})\rangle_{\mathbf{k}_F} = 122\,\text{meV}$, we extract $\tau_2 = 2\,\text{fs}$ for 1L $TaS_2$ (and $\tau_2 = 5\,\text{fs}$ for 1L $NbSe_2$, using $\Delta_{so} = 49.8\,\text{meV}$). This fit value corresponds to a rather high intervalley scattering rate, as required by the theory in order to compete against the strong SOC. The competition between these two strong effects is similar to the (possibly unphysical) balance of Ising and Rashba SOC required to fit the model of Lu *et al.* [9] to the upper critical field of superconducting electrolyte-gated $MoS_2$. It is therefore possible that in the metallic TMDs it will ultimately be necessary to simultaneously account for a combination of effects (i.e. strong SOC and one or more limiting mechanisms) in order to reconcile the difference between experiment and theory.

## Supplementary References

[1] Murphy, D. W. *et al.* Properties of $H_xTaS_2$: Correlation between the superconducting $t_c$ and an electronic instability in layer compounds. *The Journal of Chemical Physics* **62**, 967–972 (1975).
[2] Coleman, R. V., Eiserman, G. K., Hillenius, S. J., Mitchell, A. T. & Vicent, J. L. Dimensional crossover in the superconducting intercalated layer compound 2H-$TaS_2$. *Physical Review B* **27**, 125–139 (1983).
[3] Klemm, R. A. Pristine and intercalated transition metal dichalcogenide superconductors. *Physica C: Superconductivity and its Applications* **514**, 86–94 (2015).
[4] Di Salvo, F. J. A relation between the charge density wave (CDW) instability and superconducting $t_c$ in layered compounds. *Ferroelectrics* **17**, 361–364 (1977).
[5] Klemm, R. A., Luther, A. & Beasley, M. R. Theory of the upper critical field in layered superconductors. *Physical Review B* **12**, 877–891 (1975).
[6] Boyd, R. W. *Nonlinear optics* (Academic Press, 2003).
[7] Li, Y. *et al.* Probing Symmetry Properties of Few-Layer $MoS_2$ and h-BN by Optical Second-Harmonic Generation. *Nano Letters* **13**, 3329–3333 (2013).
[8] Ye, J. T. *et al.* Superconducting Dome in a Gate-Tuned Band Insulator. *Science* **338** (2012).
[9] Lu, J. M. *et al.* Evidence for two-dimensional Ising superconductivity in gated $MoS_2$. *Science* **350**, 1353–1357 (2015).
[10] Li, L. J. *et al.* Controlling many-body states by the electric-field effect in a two-dimensional material. *Nature* **529**, 185–189 (2016).
[11] Saito, Y. *et al.* Superconductivity protected by spin-valley locking in ion-gated $MoS_2$. *Nature Physics* **12**, 144–149 (2016).
[12] Xi, X., Berger, H., Forr, L., Shan, J. & Mak, K. F. Gate Tuning of Electronic Phase Transitions in Two-Dimensional $NbSe_2$. *Physical Review Letters* **117**, 106801 (2016).
[13] Lu, J. M. *et al.* A full superconducting dome of strong Ising protection in gated monolayer $WS_2$ Preprint at http://arxiv.org/abs/1703.06369 (2017).
[14] Hsu, Y.-T., Vaezi, A., Fischer, M. H. & Kim, E.-A. Topological superconductivity in monolayer transition metal dichalcogenides. *Nature Communications* **8**, 14985 (2017).
[15] Pan, J. *et al.* Enhanced Superconductivity in Restacked $TaS_2$ Nanosheets. *Journal of the American Chemical Society* **139**, 4623–4626 (2017).
[16] Sanders, C. E. *et al.* Crystalline and electronic structure of single-layer $TaS_2$. *Physical Review B* **94**, 081404 (2016).
[17] Freitas, D. C. *et al.* Strong enhancement of superconductivity at high pressures within the charge-density-wave states of 2H-$TaS_2$ and 2H-$TaSe_2$. *Physical Review B* **93**, 184512 (2016).
[18] Navarro-Moratalla, E. *et al.* Enhanced superconductivity in atomically thin $TaS_2$. *Nature Communications* **7**, 11043 (2016).
[19] Talantsev, E. F. *et al.* On the origin of critical temperature enhancement in atomically thin superconductors. *2D Materials* **4**, 025072 (2017).

[20] Xi, X. *et al.* Ising pairing in superconducting $NbSe_2$ atomic layers. *Nature Physics* **12**, 139–143 (2016).
[21] Frigeri, P. A., Agterberg, D. F., Koga, A. & Sigrist, M. Superconductivity without inversion symmetry: MnSi versus $CePt_3Si$. *Physical Review Letters* **92**, 097001 (2004).
[22] Youn, S. J., Fischer, M. H., Rhim, S. H., Sigrist, M. & Agterberg, D. F. Role of strong spin-orbit coupling in the superconductivity of the hexagonal pnictide SrPtAs. *Physical Review B* **85**, 220505 (2012).
[23] Frigeri, P. A., Agterberg, D. F. & Sigrist, M. Spin susceptibility in superconductors without inversion symmetry. *New Journal of Physics* **6**, 115–115 (2004).
[24] Fischer, M. H., Loder, F. & Sigrist, M. Superconductivity and local noncentrosymmetricity in crystal lattices. *Physical Review B* **84**, 184533 (2011).
[25] Bauer, E. & Sigrist, M. *Non-Centrosymmetric Superconductors* (Springer, 2012).
[26] Ilić, S., Meyer, J. S. & Houzet, M. Enhancement of the upper critical field in disordered transition metal dichalcogenide monolayers. *Physical Review Letters* **119**, 117001 (2017).
